# Cinnamon and Eucalyptus Oils Suppress the Inflammation Induced by Lipopolysaccharide In Vivo

**DOI:** 10.3390/molecules26237410

**Published:** 2021-12-06

**Authors:** Chen Zhao, Yuwei Cao, Zhuo Zhang, Dechao Nie, Yanling Li

**Affiliations:** Animal Science and Technology College, Beijing University of Agriculture, No. 7 Beinong Road, Changping, Beijing 102206, China; zhaochenunique@163.com (C.Z.); cyw2222021@126.com (Y.C.); Zzhuo0126@163.com (Z.Z.); niedechao0403@163.com (D.N.)

**Keywords:** cinnamon oil, eucalyptus oil, inflammation, mice

## Abstract

Inflammation caused by bacterial lipopolysaccharide (LPS) disrupts epithelial homeostasis and threatens both human and animal health. Therefore, the discovery and development of new anti-inflammatory drugs is urgently required. Plant-derived essential oils (EOs) have good antioxidant and anti-inflammatory activities. Thus, this study aims to screen and evaluate the effects of cinnamon oil and eucalyptus oil on anti-inflammatory activities. The associated evaluation indicators include body weight gain, visceral edema coefficient, superoxide dismutase (SOD), glutathione peroxidase (GSH-Px), malondialdehyde (MDA), nitrogen monoxide (NO), interleukin-6 (IL-6), interleukin-10 (IL-10), tumor necrosis factor alpha (TNF-α), Urea, Crea, ALT, TLR4, MyD88, NF-κB, IκB-α, iNOS, and Mn-SOD. In addition, tissue injury was determined by H&E staining. The results revealed that cinnamon oil and eucalyptus oil suppressed inflammation by decreasing SOD, TNF-α, and NF-κB levels. We also found that cinnamon oil increased the level of GSH-Px, MDA, and Mn-SOD, as well as the visceral edema coefficient of the kidney and liver. Altogether, these findings illustrated that cinnamon oil and eucalyptus oil exhibited wide antioxidant and anti-inflammatory activities against LPS-induced inflammation.

## 1. Introduction

Inflammation is usually considered as the complex immune responses to defend against various pathogens, toxic compounds, and environmental stress factors, but excessive inflammation is harmful to the host [1]. In addition, self-damage caused by inflammation is inevitable, and if it cannot be resolved in time, it will lead to pathological conditions [2]. If the inflammation subsides insufficiently or the stimulation persists, it may lead to the continuous activation of immune cells and damage the health of the body [3]. Nonsteroidal and glucocorticoid drugs are mainly used to treat inflammation; however, they have many adverse reactions and are not suitable for long-term use. Moreover, many countries have banned antibiotic growth promoters for animal growth promotion [4]. Therefore, the appearance of extracts derived from plants is of great significance to inflammation.

Essential oils (EOs), as a kind of plant-derived extracts, are a kind of secondary metabolites that are derived from natural plants [5]. EOs are usually used for the prevention and treatment of various diseases, such as acute lung injury, asthma, and so on. Their unique medicinal properties, such as anti-inflammatory, antibacterial, and antioxidant activities, have been confirmed by many published papers [6]. The antibacterial effect of EOs is generally due to their lipophilic properties and ability to penetrate the cell wall and plasma membrane, which destroys the membrane structure of bacteria [7]. Limonene [8], *Cinnamomum camphora* (L.) J. Presl essential oil [9], and ginger volatile oil [10] have been proven to have a correlation between their anti-inflammatory properties and their ability to inhibit proinflammatory cytokines. Inhibiting the inappropriate production of inflammatory mediators can prevent or improve inflammatory diseases. Furthermore, EOs have shown considerable potential for inflammatory treatment over the past century, incuding lemon essential oil [11], oregano essential oil [12,13], and so on. Many countries are increasingly banning the use of antibiotics as feed additives in livestock feed, and EOs have gradually attracted people’s attention [14]. Based on the previous screening of the antioxidant activities of different essential oils, eucalyptus oil and cinnamon oil had good antioxidant activity in vitro [15,16,17]. Therefore, eucalyptus oil and cinnamon oil were selected for subsequent experiments. In the present study, we aimed to investigate the preventive effect of essential oils regarding anti-inflammatory properties using the LPS-induced mice model. We have a new understanding of their anti-inflammatory mechanisms.

In order to assess the anti-inflammatory activity of EOs and explore the underlying mechanisms, this study was designed to detect the weight of LPS-induced mice, the production of antioxidant indexes (SOD, GSH-Px, and NO), inflammatory cytokines secretion (IL-6, IL-10, TNF-α), serum metabolic indexes (Urea, Crea, and ALT), and mRNA expression of TLR4, MyD88, NF-κB, IκB-α, iNOS, and Mn-SOD. In this study, we explored whether pretreatment with cinnamon oil and eucalyptus oil could alleviate the damage of LPS-induced mice, respectively. We hope to provide better insights into the anti-inflammatory potential of EOs.

## 2. Results

### 2.1. LD50 of EOs

According to the modified Karber’s method, the LD50 of mice with oral administration of cinnamon oil was 2979 mg/kg, with a 95% confidence interval of 2590~3424 mg/kg; the LD50 of mice with oral administration of eucalyptus oil was 3065 mg/kg, with a 95% confidence interval of 2721~3453 mg/kg (Table 1).

### 2.2. Effects of EOs on Body Weight Gain and Visceral Edema Coefficient

In order to detect the effect of EOs on the body weight of LPS-induced mice, we weighed mice one week and two weeks after LPS stimulation, and weighed the organs after autopsy. The results are shown in Table 2. LPS significantly elevated the body weight loss (*P* < 0.05) compared with the normal saline group. Cinnamon oil and eucalyptus oil at doses of 250 mg/kg, 500 mg/kg, 750 mg/kg, and 1000 mg/kg had no significant effect on body weight gain compared with the LPS group. LPS (10 mg/kg) significantly elevated the visceral edema coefficient (kidney, *P* < 0.05; and liver, *P* < 0.05) compared with the normal saline group. Cinnamon oil (750 mg/kg and 1000 mg/kg) notably decreased the visceral edema coefficient (kidney and liver, *P* < 0.05) compared with the LPS group. Moreover, cinnamon oil at a dose of 500 mg/kg significantly decreased the coefficient of kidney edema (*P* < 0.05) compared with the LPS group. Eucalyptus oil at doses of 250 mg/kg, 500 mg/kg, 750 mg/kg, and 1000 mg/kg had no significant effect on the visceral edema coefficient compared with the LPS group.

### 2.3. Effect of EOs on Antioxidant Index in Serum of Mice Induced by LPS

**Figure 1:**
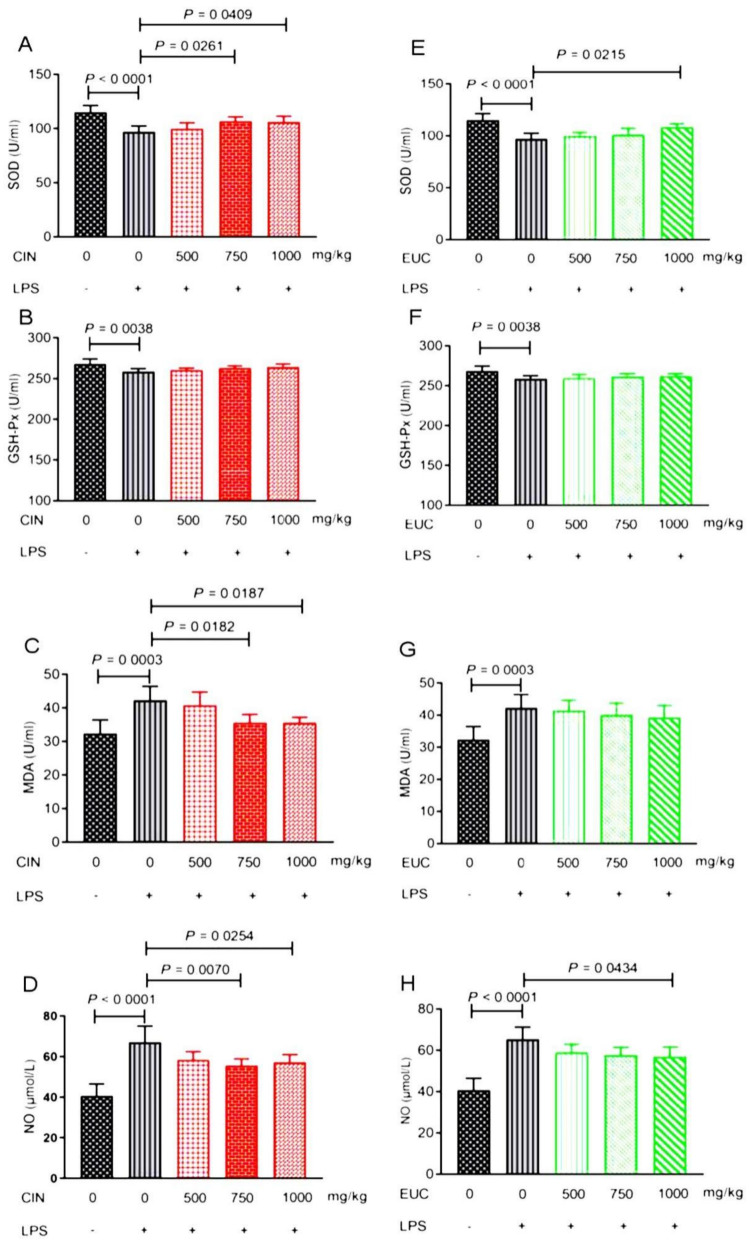
Effect of EOs on antioxidant indexes of serum in mice induced by LPS. Effects of cinnamon oil on LPS-induced production of superoxide dismutase (SOD) (**A**), glutathione peroxidase (GSH-Px) (**B**), malondialdehyde (MDA) (**C**), and nitric oxide (NO) (**D**) in mice. Effects of eucalyptus oil on LPS-induced production of SOD (**E**), GSH-Px (**F**), MDA (**G**), and NO (**H**) in mice. Data are presented as mean ± standard error of measurement (mean ± SEM) (*n* = 6). The concentration of LPS induced in mice was 10 mg/kg. The concentration of cinnamon oil and eucalyptus oil were 500 mg/kg, 750 mg/kg, and 1000 mg/kg, respectively. CIN—cinnamon oil; EUC—eucalyptus oil.

In order to investigate the antioxidant effect of EOs, SOD, GSH-Px, MDA, and NO production were evaluated in LPS-induced mice. As shown in Figure 1, LPS caused significantly decreased levels of SOD and GSH-Px in serum of mice, while it increased the levels of MDA and NO in serum of mice compared with the normal saline group. Conversely, pretreatment with cinnamon oil efficiently increased the level of SOD (Figure 1A), reduced the level of MDA (Figure 1C) and NO (Figure 1D), and had no influence on GSH-Px (Figure 1B). Pretreatment with eucalyptus oil increased the level of SOD (Figure 1E), reduced the level of NO (Figure 1H), and had no influence on the changes of GSH-Px (Figure 1F) and MDA (Figure 1G). Cinnamon oil and eucalyptus oil could play an antioxidant role by changing the content of antioxidant indexes in LPS-induced mice, and cinnamon oil had a better effect.

### 2.4. Effect of EOs on IL-6, IL-10, and TNF-α in Serum of LPS-Induced Mice

**Figure 2:**
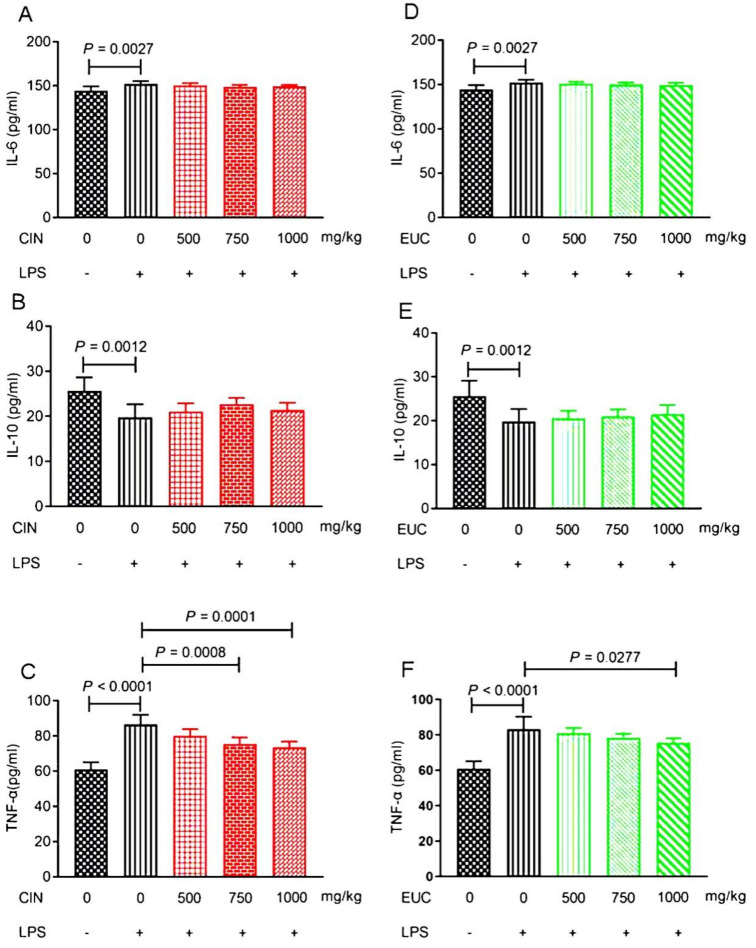
Effect of EOs on inflammatory factors of serum in mice induced by LPS. Effects of cinnamon oil on LPS-induced secretion of IL-6 (**A**), IL-10 (**B**), and TNF-α (**C**) in mice. Effects of eucalyptus oil on LPS-induced secretion of IL-6 (**D**), IL-10 (**E**), and TNF-α (**F**) in mice. Data are presented as mean ± standard error of measurement (mean ± SEM) (*n* = 6). The concentration of LPS induced in mice was 10 mg/kg. The concentration of cinnamon oil and eucalyptus oil were 500 mg/kg, 750 mg/kg, and 1000 mg/kg, respectively. CIN, cinnamon oil; EUC, eucalyptus oil.

To confirm the anti-inflammatory effect of EOs, the levels of inflammatory factors, including IL-6, IL-10, and TNF-α were evaluated in LPS-stimulated mice. Compared with the control group, LPS increased the secretion of IL-6 and TNF-α, and decreased the secretion of IL-10 compared with the normal saline group (Figure 2A–F). The results indicated that the pretreatment with cinnamon oil and eucalyptus oil only could reduce the secretion of TNF-α compared with the LPS group (Figure 2C,F).

### 2.5. Effect of EOs on Urea, Crea, and ALT in Serum of LPS-Induced Mice

As shown in Table 3, the LPS group significantly increased the level of ALT compared with the normal saline group (*P* < 0.05). Pretreatment with cinnamon oil alone could reduce the content of ALT in a concentration-dependent manner. However, there was no significant difference in the levels of Urea, Crea, and ALT under the pretreatment with eucalyptus oil.

### 2.6. Effect of EOs on the Expression of Liver-Related Genes in LPS-Induced Mice

**Figure 3:**
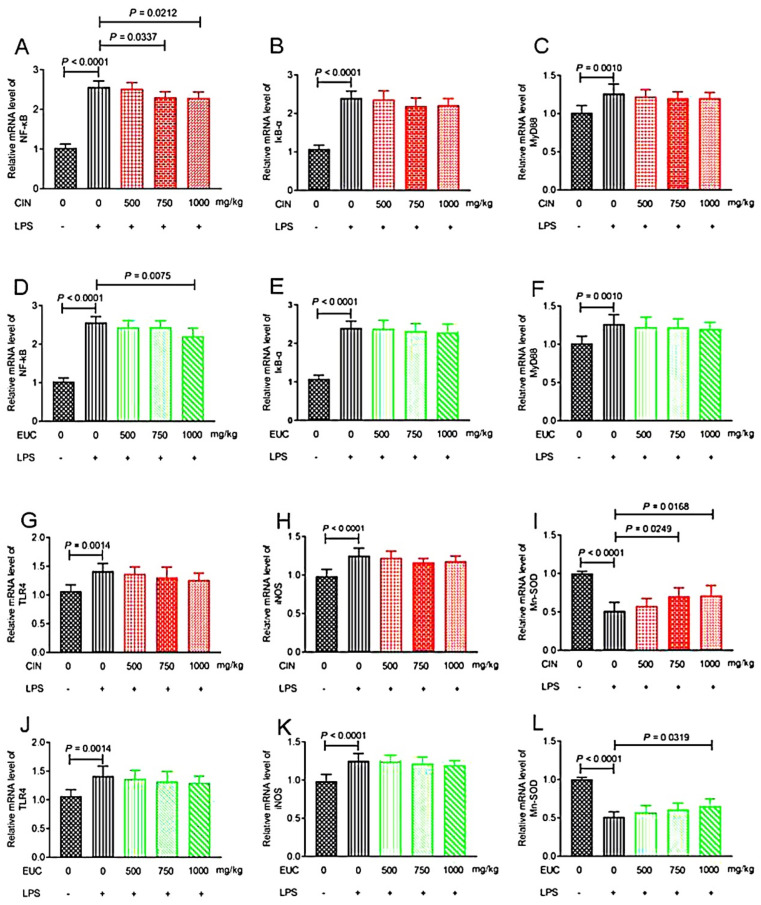
The effect of EOs on the expression of inflammation-related genes in the liver. Effects of cinnamon oil on LPS-induced mRNA expression levels of NF-κB (**A**), IκB-α (**B**), MyD88 (**C**), TLR4 (**G**), iNOS (**H**), and Mn-SOD (**I**) in mice. Effects of eucalyptus oil on LPS-induced mRNA expression levels of NF-κB (**D**), IκB-α (**E**), MyD88 (**F**), TLR4 (**J**), iNOS (**K**), and Mn-SOD (**L**) in concentration of LPS induced in mice was 10 mg/kg. The concentration of cinnamon oil and eucalyptus oil were 500 mg/kg, 750 mg/kg, and 1000 mg/kg, respectively. CIN—cinnamon oil; EUC—eucalyptus oil.

To investigate the anti-inflammatory effect of EOs, we examined the levels of inflammatory mediators, including NF-κB, IκB-α, MyD88, TLR4, iNOS, and Mn-SOD. Compared with the normal saline group, LPS increased NF-κB, IκB-α, MyD88, TLR4, and iNOS in liver significantly, and decreased Mn-SOD (Figure 3A–L). Conversely, pretreatment with cinnamon oil decreased the production of NF-κB (Figure 3A), and increased the production of Mn-SOD (Figure 3I). Pretreatment with eucalyptus oil decreased the production of NF-κB (Figure 3D), and increased the production of Mn-SOD (Figure 3L).

## 3. Discussion

LPS, as a Gram-negative bacterial constituent [18], is one of the most important pathogenic molecules that mediate the inflammatory damage of acute infection. Many diseases, such as bronchitis, pneumonia, and hepatitis, are closely related to LPS-induced inflammation [19]. In this study, it was found that 12 h after 10 mg/kg LPS stimulation in mice, there was sparse coat, mental depression, secretion around the corner of the eye and anus, and even death. After dissection, the surface of the liver was white, swollen, and round. Therefore, we chose 10 mg/kg LPS to stimulate mice. We further studied the protective effect of the pretreatment with EOs on mice stimulated by LPS.

As shown in Table 2, we found that the weight of mice decreased significantly 12 h after LPS stimulation compared with the normal saline group. The pretreatment of eucalyptus oil had no negative effect on the weight of mice, while the pretreatment of cinnamon oil could effectively inhibit the weight loss of mice stimulated by LPS. In general, the coefficient of visceral edema is applicable to the liver, kidney, spleen, and other substantive organs. If the coefficient is larger, it indicates that there may be edema, hemorrhage, and hyperplasia [20]. Previous reports showed that patchouli oil could reduce pulmonary edema [21]. Pretreatment with pogostone evidently reduced the lung W/D ratio, and the magnitude of pulmonary edema was quantified by the lung W/D weight ratio [22]. In this study, LPS stimulation in mice could cause significant kidney edema and liver edema, while cinnamon oil could reduce the liver edema in mice induced by LPS at the concentrations of 750 mg/kg and 1000 mg/kg. Eucalyptus oil did not show the effect of alleviating visceral edema in mice induced by LPS.

When stimulated by exogenous substances, it will break the balance and lead to oxidative stress. The changes of antioxidant enzymes, free radicals, and oxidation products can effectively reflect the degree of oxidative damage [23]. SOD is the primary substance for scavenging free radicals in vivo [24], which can disproportionate it to H_2_O_2_ by catalyzing •O^2−^, and weaken lipid peroxidation [25]. In this study, cinnamon oil and eucalyptus oil could significantly increase the level of SOD in serum of mice compared with the LPS group. GSH-Px is a general term for peroxidase active enzymes, which can reduce H_2_O_2_ to H_2_O to prevent liver injury [26]. Cinnamon oil could significantly increase the level of GSH-Px in serum of mice compared with the LPS group; on the other hand, pretreatment with eucalyptus oil showed no changes. Lipid peroxidation is one of the markers of oxidative stress, and MDA is one of the end products of tissue lipid peroxidation. The higher the level of MDA, the more serious the oxidative damage [27]. Cinnamon oil (750 and 1000 mg/kg) could significantly reduce MDA in serum of mice. Bellassoued et al. [28] found that Mentha piperita leaf essential oil can increase the levels of SOD and GSH-Px in the plasma and liver of rats induced by CCl_4_, and reduce the level of MDA. NO, as a toxic active nitrogen free radical produced by iNOS, can scavenge pathogens. However, it will cause oxidative damage to the body, and then cause inflammation when it is excessive. Therefore, to some extent, both cinnamon oil and eucalyptus oil may eliminate free radicals, delay the consumption of SOD and GSH-Px activities, and reduce the level of MDA in vivo, so as to further alleviate the oxidative damage in mice induced by LPS. However, cinnamon oil may have great potential, which can effectively influence the change of oxidation index at low concentration. Cinnamon oil and eucalyptus oil might enhance antioxidant status by changing the level of antioxidant factors and reducing lipid peroxidation. Moreover, cinnamon oil and eucalyptus oil had a substantial protective effect on mice stimulated by LPS.

When stimulated with LPS, activation of macrophages plays an important role in the initiation and propagation of inflammatory responses by the production of cytokines, IL-1β, TNF-α, and other inflammatory mediators [29]. TNF-α is an important mediator of endotoxin toxicity and the first inflammatory mediator to induce excessive inflammatory reaction [30]. In addition, TNF-α has a wide range of important biological effects and participates in the inflammatory response together with other cytokines such as interleukin and interferon [31]. Thus, EOs can protect the inflammatory response induced by LPS by inhibiting the production of inflammatory mediators. Previous studies have shown that cinnamaldehyde can inhibit the production of IL-1β and TNF-α by mouse j774A.1 macrophages, and can effectively reduce the release of ROS induced by LPS, which confirms the antioxidant and anti-inflammatory properties of cinnamaldehyde, which provides a possibility for the application of cinnamaldehyde in the field of immune regulation [32]. Moreover, previous studies on the anti-inflammatory activity of cinnamaldehyde in vitro showed that cinnamaldehyde could inhibit the production of IL-1β and TNF-α by J774A.1 macrophages and THP-1 monocytes induced by LPS, and inhibit the secretion of pre IL-1β by THP-1 monocytes induced by LPS [33]. Our results were consistent with the above, and cinnamon oil and eucalyptus oil could reduce the level of TNF-α to protect mice induced by LPS. IL-6 is a pro-inflammatory cytokine, which can induce neutrophil apoptosis, regulate the expression of macrophage colony-stimulating factor receptor, and promote the differentiation of macrophages [34]. IL-10 is an anti-inflammatory cytokine, which can inhibit the activation of NF-кB and the release of pro-inflammatory cytokines by macrophages [35]. When the concentration of essential oil was 200 μg/mL, the anti-inflammatory effect of the essential oil extracted from H. Sabdariffa might be through inhibiting the activation of NF-κB and MAPKs signal pathway and inhibiting the production of proinflammatory cytokines (IL-1, IL-6, TNF-α, COX-2, and iNOS) [36]. Toden et al. [37] studied the anti-inflammatory mechanism of essential turmeric oil–curcumin (ETO-curcumin), and found that ETO-curcumin can up-regulate anti-inflammatory cytokines such as IL-10 and IL-11, and inhibit chemokine CCL17. IL-1β, IL-6, and TNF-α were mainly produced by activated monocytes or macrophages. Oregano essential oil (2.5–10 µg/mL) inhibited the expression and secretion of IL-1β, IL-6, and TNF-α in RAW264.7 cells treated with LPS (1 µg/mL), and it had a protective effect on inflammatory response [38]. It was observed that citral pretreatment could significantly inhibit the production of TNF-α, IL-6, IL-1β, and NF-κB in rats with acute lung injury induced by LPS in vivo and in vitro [39]. The results of enzyme immunoassay showed that Abies koreana essential oil could significantly inhibit the release of IL-1β, IL-6, and TNF-α in RAW264.7 cells induced by LPS, and Abies koreana essential oil had a strong inhibitory effect on pro-inflammatory mediators [40]. Artemisia princeps Pamp. (family Asteraceae) essential oil could inhibit the expressions of pro-inflammatory cytokines (IL-1β, IL-6, TNF-α) and iNOS and the activation of NF-κB, and increase expression of the anti-inflammatory cytokine IL-10 [41]. The essential oil from Artemisia argyi could significantly inhibit NO and PGE2, and strongly inhibit IL-6, IL-10, IFN-β, and monocyte chemoattractant protein-1 (MCP-1), but the inhibitory effect on TNF-α is relatively weak in 264.7 macrophages stimulated by LPS [42]. Essential oil from Citrus aurantium L. has been recently reported to inhibit NO, IL-6, TNF-α, and IL-1β production, as well as their gene expression level in LPS-stimulated RAW264.7 cells [43]. Our results were inconsistent with the above studies. In our study, cinnamon oil and eucalyptus oil had no effect on the decrease of IL-6 level and the increase of IL-10 level in mice induced by LPS. Consistent with our results, Kutlu et al. [44] showed that essential oil obtained from the fruit of myrtus communis L. and the compound α-terpineol had no effect on the decreased cell index and increased cytokine response due to LPS-induced endothelial cell damage. The inhibitory effects of some essential oils, including thyme, oregano, Artemisia fukudo, and half-ripe chinotto, have been reported to affect the production of proinflammatory mediators NO, IL-1β, and IL-6, which are mainly regulated by transcription [45,46,47].

The levels of Urea and Crea in serum can reflect renal function, while AST and ALT can be used to evaluate liver function. The LPS group had no significant effect on Urea and Crea compared with the normal saline group. It was speculated that LPS didn’t cause renal injury in mice. The ALT content in liver was significantly increased in the LPS group compared with the normal saline group (*P* < 0.01). After LPS stimulation, the liver of mice was damaged. Cinnamon oil and eucalyptus oil had no significant effect on Urea and Crea compared with the LPS group. However, cinnamon oil could effectively reduce the content of ALT at the concentrations of 750 mg/kg and 1000 mg/kg. In addition, the result showed that only cinnamon oil alleviated the liver injury in mice induced by LPS. Uchida et al. [48] elucidated citral’s effect in preventing acetaminophen-induced acute toxicity with the ability to reduce the serum levels of hepatic enzymes (AST, ALT, alkaline phosphatase, and gamma-glutamyltransferase), decrease oxidative stress, and demonstrate an anti-inflammatory role in the hepatic tissue. Rašković et al. [49] found that 5 mg/kg and 10 mg/kg rosemary essential oil could reduce the activities of ALT and AST in serum of rats with acute liver injury induced by carbon tetrachloride by 2 times, and 10 mg/kg rosemary essential oil would lead to a statistically significant decrease in urea and crea levels. These results indicated that rosemary essential oil had the ability of liver protection and recovery of renal excretion function. Bellassoued et al. [50] found that pretreatment with essential oil of cinnamomum verum (70 and 100 mg/kg) prior to CCl_4_ injection prevented the rise of ALT, AST, urea, and crea compared with the CCl_4_-treated rats. In addition, 150 ppm and 200 ppm essential oil blends of thyme, peppermint, and eucalyptus (40:40:20) could decrease Alt, AST, and serum alkaline phosphatase in the liver of broilers (*P* < 0.05) [51]. Our results are inconsistent with the above results. We made the following conjectures: the pretreatment dose of EOs was low enough to change the content of ALT, urea, and crea, or the action time of EOs was short. However, EOs didn’t aggravate the liver damage of mice caused by LPS, and it was also a protective effect to some extent.

In resting cells, NF-κB is sequestered in the cytoplasm, bound by its inhibitor IκB-α. Once stimulated by LPS, the degradation and phosphorylation of IκB-α will increase, which results in the release of free NF-κB p65 and translocation from the cytoplasm to the nucleus, triggering the transcription of specific target genes such as TNF-α, IL-6, and IL-1β [52,53,54,55]. NF-κB is an important transcription factor required for expression of many cytokines involved in inflammation stimulated by LPS [56]. Thus, treatment aiming at inhibiting the NF-κB signal pathway may be a logical therapeutic target for inflammation. The results showed that 1,8-cineole could inhibit nuclear NF-κB p65 translocation through IκB-α, thus reducing the level of proinflammatory NF-κB target gene and regulating the inflammatory signal pathway [57]. It can also inhibit Mn-SOD, activate the activity of iNOS, and promote the release of NO by immune leukocytes. iNOS is responsible for the production of NO, and once induced by LPS, the TLR-4 inflammatory pathway is activated, which increases the production of inflammatory mediators, such as NO [58]. Gonçalves et al. [59] have recently reported the anti-inflammatory and antihyperalgesic activities of citral through the modulation of TLR-4, TLR-2/dectin-1 ligands, and cannabinoid receptor 2 (CB2R) in mice induced by LPS. In mice induced by LPS, the anti-inflammatory effect of cinnamaldehyde was related to the inhibition of TLR4/MD2, MyD88, NLRP3, ASC, and caspase-1 expression, but not inhibition of NF-κB activation and caspase-1 activity [60]. S. impressa essential oil could explain the significant decrease of nitrite level by significantly reducing the expression of iNOS in macrophages stimulated by LPS, thus enhancing the anti-inflammatory potential [61]. D-limonene, as the main component of citrus essential oil, effectively decreased the overexpression of NF-κB, COX-2, and iNOS and NO in the kidney of Wistar rats to inhibit the inflammatory reaction [62]. In rats, cinnamaldehyde inhibited senescence related NF-κB activation and subsequent iNOS and cyclooxygenase expression by improving oxidative-stress-related signaling pathways [63]. In this study, cinnamon oil and eucalyptus oil may inhibit the separation of IκB-α and NF-κB, thus inhibiting the entry of NF-κB into the nucleus, regulating the expression of inflammatory-factor-related genes and reducing the release of inflammatory cytokines. At the same time, some cytokines, such as TNF-α, can activate the NF-κB signaling pathway, increase the activity of SOD, and inhibit iNOS, so as to increase the antioxidant effect and reduce the production of free radicals such as NO and ROS. In the presence of essential oil, the decrease of NO may be due to the direct scavenging effect of essential oil on NO, or the inhibition of iNOS expression [61]. Xu et al. [64] showed that essential oil of schisandra chinensis decreased the level of NO and inhibited the activation of MAPKs in BV-2 microglia stimulated by LPS, which suggested that the essential oil may be mediated by the NF-κB/MAPK signaling pathway, which regulates neuroinflammation. Illicium anisatum essential oil (25, 50, and 100 mg/mL) significantly inhibited the production of NO and iNOS in a dose-dependent manner in RAW 264.7 cells induced by LPS, and Illicium anisatum essential oil positively inhibited the expression of inflammatory-related factors [65]. Studies showed that intraperitoneal injection of cinnamaldehyde can improve the survival rate of inflammatory mice, which is related to the inhibition of the TLR-4-NF-κB signal transduction pathway [66]. Previous literature has revealed that the anti-inflammatory activities of several plants of the genus cinnamomum are due to the inhibitory activity of cytokine including PGE2, NO, TNF-α, IL-1β, IL-12, and IL-6 [67,68,69,70]. Therefore, EOs could reduce oxidative stress and liver injury by changing the content of related factors in mice induced by LPS, so as to slow down the occurrence of inflammation.

## 4. Materials and Methods

### 4.1. Chemicals and Reagents

LPS (L2880) was purchased from Sigma (St. Louis, MO, USA). TNF-α, IL-6, and IL-10enzyme-linked immunosorbent assay (ELISA) kit were provided by Beijing SINO-UK Institute of Biological Technology (Beijing, China). All other reagents were of analytical grade.

### 4.2. Essential Oils

Two kinds of essential oils (EOs) were selected, cinnamon oil and eucalyptus oil. The purity of them was 80%. They were purchased from Nanjing Vincero International Trade Co., Ltd. (Nanjing, China). 

### 4.3. Animals

Five-week male Kunming mice (22.0 ± 1.5 g, *n* = 160) were purchased from SPF Biotechnology Co., Ltd. (Beijing, China). Animals were housed under conditions of temperature of 24 ℃ and humidity of 40–80%, with food and water provided ad libitum. All experimental procedures were approved by the Laboratory Animal Ethics Committee of Beijing University of Agriculture and conformed to the legal mandates and national guidelines for the care and maintenance of laboratory animals (SYXK(京)2021-0001, 4 January 2021).

### 4.4. Acute Toxicity Test of EOs

Mice were randomly divided into 8 groups, with 10 mice in each group, half male and half female. The mice were given 1250, 2500, 3750, and 5000 mg/kg of cinnamon oil and eucalyptus oil by gavage, respectively. The survival rate of mice for 2 weeks was observed and recorded. LD50 of EOs was calculated by modified Karber’s method.

### 4.5. Experimental Designs

The mice were randomly divided into six groups (*n* = 6 per group) for each EO (cinnamon oil and eucalyptus oil) as follows: (a) negative control group (normal saline); (b) positive control group (LPS, 10 mg/kg); (c) LPS + cinnamon oil group (500, 750, and 1000 mg/kg); (d) LPS + eucalyptus oil group (500, 750, and 1000 mg/kg). All mice were given EO by oral gavage for two weeks, except those in the negative and positive control group, which were given normal saline by oral gavage. After 6 h of the last administration, all mice were injected intraperitoneally with LPS, except those in the negative control group, which were injected intraperitoneally with normal saline. Mice were sacrificed, and samples were collected at 12 h after LPS administration.

### 4.6. Calculation of Body Weight Gain and Visceral Edema Coefficient

Calculation of body weight gain: The initial body weight, and body weight after 1 week, 2 weeks, and 12 h after LPS stimulation were recorded. The body weight gain was calculated for 0–7 days, 7–14 days, and after LPS stimulation.

Visceral edema coefficient: Fresh kidneys, spleens, livers, and lungs were taken, and their weights were measured and recorded. The coefficient of kidney edema in percent (I%) was calculated as follows: I% = [mk/(mk + ms + mli + mlu)] × 100%. According to the formula, mk is the weight of kindey, ms is the weight of spleen, mli is the weight of liver, and mlu is the weight of lung.

### 4.7. Enzyme-Linked Immune Sorbent Assay (ELISA)

The blood samples were extracted from eyeballs of mice, and centrifuged at 3500 rpm for 10 min to obtain the supernatant. All the serum samples were stored at −80 ℃ for ELISA. The content levels of inflammatory cytokines including tumor necrosis factor alpha (TNF-α), interleukin 6 (IL-6), and interleukin 10 (IL-10) in blood serum were measured. The content levels of antioxidant index including superoxide dismutase (SOD), glutathione peroxidase (GSH-Px), and nitrogen monoxide (NO) in blood serum were measured. The content levels of metabolic index including tumor necrosis urea, creatinine (crea), and alanine aminotransferase (ALT) in blood serum were measured. All indicator levels were quantified by ELISA kits (Beijing SINO-UK Institute Of Biological Technology, China) according to the manufacturer’s instructions.

### 4.8. RT-PCR

Fresh liver tissue (100 mg) was stored in liquid nitrogen for RT-PCR. The extraction of total RNA concentration was measured using RNA simple total RNA kit (DP431, Beijing, China) according to the manufacturer’s instructions. The concentration and integrity of total RNA were measured by micro ultraviolet spectrophotometer with A260 nm/A280 nm as the standard. The first strand synthesis kit of Fast Quant cDNA (KR106-02, Beijing, China) was used for the cDNA synthesis, according to the manufacturer’s protocol. The program of real-time fluorescent quantitative PCR was carried out according to Super Real fluorescent quantitative pre-mixing kit (FP205-01, Beijing, China). The PCR primers were designed by Shanghai Bioengineering Co., Ltd. (Shanghai, China), and the primer sequences are shown in Table 4. qRT-PCR was performed on the Stratagene Mx3005p Sequence Detection System (Agilent Technologies, Waldbronn, Germany) using the SYBR Green real-time master mix. The relative gene expression was quantified by the comparative 2^−∆∆CT^ method.

### 4.9. Statistical Analysis

The results obtained were expressed as the mean ± SEM. Excel 2017 was used for preliminary sorting of the data, and the generalized linear model (GLM) of Statistics Analysis System (SAS) 9.4 was used for statistical analysis. Statistical significance was assessed in comparison with the respective control for each experiment using one-way analysis of variance. A *P* value less than 0.05 indicated a statistically significant difference.

## 5. Conclusions

In summary, our findings demonstrated the protective effects of cinnamon oil and eucalyptus oil on LPS-induced mice inflammation, paving the way for rational use of essential oils in inflammatory diseases. They also provided the basis for the pretreatment of essential oils in animal husbandry.

## Figures and Tables

**Table 1:** Acute oral toxicity of EOs in mice.

EOs	Group	Dose (mg/kg)	Mortality (%)	Survival (%)
CIN	1	1250.00	0.10	0.90
	2	2500.00	0.50	0.50
	3	3750.00	0.70	0.30
	4	5000.00	1.00	0.00
EUC	1	1250.00	0.00	1.00
	2	2500.00	0.40	0.60
	3	3750.00	0.80	0.20
	4	5000.00	1.00	0.00

**Table 2:** Effects of EOs on weight and viscera of mice induced by LPS.

Item		Control	LPS	Dose (mg/kg)			SEM	*P*		
				500	750	1000		*P* ^1^	*P*_L_	*P*_Q_
1 week’s weight gain (g)	CIN	7.9	7.7	7.2	7.3	7.1	0.25	0.08	0.09	0.61
	EUC	7.9	7.7	7.2	7.4	7.0	0.32	0.16	0.16	0.92
2 week’s weight gain (g)	CIN	16.1	15.7	15.1	15.2	15.4	0.30	0.26	0.33	0.17
	EUC	16.1	15.7	15.1	14.9	15.3	0.32	0.14	0.19	0.15
Weight gain after LPS induced (g)	CIN	−2.20	−2.73 ^#b^	−2.60 ^ab^	−2.47 ^ab^	−2.44 ^ab^	0.09	0.04	0.03	0.93
	EUC	−2.20	−2.73 ^#^	−2.63	−2.52	−2.46	0.10	0.05	0.05	0.84
Kidney (g)	CIN	1.32	1.43 ^#^	1.41	1.37	1.38	0.02	0.13	0.07	0.77
	EUC	1.32	1.43 ^#^	1.42	1.39	1.37	0.03	0.20	0.09	0.53
Spleen (g)	CIN	0.46	0.52	0.51	0.50	0.51	0.02	0.87	0.54	0.73
	EUC	0.46	0.52	0.50	0.51	0.49	0.02	0.59	0.24	0.97
Liver (g)	CIN	5.94	6.47 ^#a^	6.40 ^ab^	6.19 ^bc^	6.16 ^bc^	0.11	0.02	<0.01	0.60
	EUC	5.94	6.47 ^#a^	6.42 ^a^	6.35 ^a^	6.31 ^ab^	0.11	0.02	0.12	0.78
Lung (g)	CIN	0.81	0.88	0.86	0.84	0.85	0.02	0.46	0.19	0.60
	EUC	0.81	0.88	0.86	0.85	0.84	0.03	0.68	0.29	0.99
Total viscera (g)	CIN	8.52	9.32 ^##a^	9.16 ^ab^	8.88 ^bc^	8.88 ^bc^	0.11	<0.01	<0.01	0.94
	EUC	8.52	9.32 ^##a^	9.20 ^a^	9.09 ^a^	9.00 ^ab^	0.11	<0.01	0.03	0.79

*P*^1^ is the value of the effect of LPS group and essential oil group on the weight gain of LPS-induced inflammatory body in mice. Different superscript letters (a, b, c) in the same line indicate that EOs group was significantly different compared with LPS group; *P*_L_ and *P*_Q_ are the values of linear and quadratic curve effects of LPS group and EOs group (500, 750, and 1000 mg/kg). Control—normal saline group; CIN—cinnamon oil; and EUC—eucalyptus oil; SEM—standard error of measurement. ^#^ *P* < 0.05, and ^##^ *P* < 0.01, compared with the control group.

**Table 3:** Effects of EOs on serum metabolic indexes of mice induced by LPS.

Item		Control	LPS	Dose (mg/kg)			SEM	*P*		
				500	750	1000		*P* ^1^	*P*_L_	*P*_Q_
Urea (mmol/L)	CIN	23.2	24.5	24.3	23.9	24.0	0.52	0.74	0.44	0.93
	EUC	23.2	24.5	24.4	24.2	23.9	0.58	0.75	0.50	0.77
Crea (umol/L)	CIN	46.1	50.2	49.5	48.5	47.9	1.11	0.36	0.15	0.81
	EUC	46.1	50.2	49.7	49.0	48.5	1.19	0.47	0.33	0.86
ALT (U/L)	CIN	17.4	22.5 ^##a^	21.7 ^ab^	20.1 ^bc^	19.8 ^bc^	0.77	0.02	0.01	0.75
	EUC	17.4	22.5 ^##a^	21.9 ^a^	21.1 ^ab^	20.4 ^ab^	0.73	0.02	0.05	0.66

*P*^1^ is the value of the effect of LPS group and essential oil group on the weight gain of LPS−induced inflammatory body in mice. Different letters in the same line indicate that EOs group was significantly different compared with LPS group; *P*_L_ and *P*_Q_ are the values of linear and quadratic curve effects of LPS group and EOs group (500, 750, and 1000 mg/kg); Control, normal saline group; CIN—cinnamon oil; and EUC—eucalyptus oil; SEM—standard error of measurement. ^##^ *P* < 0.01, compared with the control group.

**Table 4:** Sequence information on the primers used for quantitative PCR.

Items	Sequences	Products/bp
TLR4	F: GCCATCATTATGAGTGCCAATT	107
	R: AGGGATAAGAACGCTGAGAATT	
MyD88	F: CGGAACTTTTCGATGCCTTTAT	107
	R: CACACACAACTTAAGCCGATAG	
NF-κβ p65	F: TCCAGGCTCCTGTTCGAGTCTC	106
	R: CGGTGGCGATCATCTGTGTCTG	
IκB-α	F: CTGGTTTCGCTCTTGTTGAAAT	150
	R: GGGTAGCATCTGGAGATTTTCC	
iNOS	F: TGCCACGGACGAGACGGATAG	109
	R: CTCTTCAAGCACCTCCAGGAACG	
Mn-SOD	F: AAGGGAGATGTTACAACTCAGG	96
	R: GCTCAGGTTTGTCCAGAAAATG	
β-actin	F: CTACCTCATGAAGATCCTGACC	90
	R: CACAGCTTCTCTTTGATGTCAC	

## Data Availability

Data is contained within the article.

## Sample Availability

Samples of the compounds are available from the authors.

## Abbreviation

ALT	Alanine aminotransferase
Crea	Creatinine
EOs	Essential oils
GSH-Px	Glutathione peroxidase
IκB-α	Inhibitor of NF-κB
IL-6	Interleukin-6
IL-10	Interleukin-10
iNOS	Inducible nitric oxide synthase
LPS	lipopolysaccharide
MDA	Malondialdehyde
Mn-SOD	Manganese superoxide dismutase
MyD88	Myeloid differentiation factor 88
NF-κB	Nuclear factor kappa-B
NO	Nitric oxide
SOD	Superoxide dismutase
TLR4	Toll-like receptor 4
TNF-α	Tumor necrosis factor alpha
Urea	Urea
